# Understanding the Role of Toll-Like Receptors 9 in Breast Cancer

**DOI:** 10.3390/cancers16152679

**Published:** 2024-07-27

**Authors:** Umaima Al-alem, Alaa Al-Saruri, Hasan Bamahros, Abeer M. Mahmoud, Emily Sible, Uzma A. Hasan

**Affiliations:** 1 Division of Epidemiology and Biostatistics, School of Public Health, University of Illinois at Chicago, Chicago, IL 60607, USA; 2 Department Psychologie, Friedrich-Alexander-Universität Erlangen-Nürnberg, 91058 Erlangen, Germany; alaa.r.al-saruri@fau.de; 3 College of Business Administration, University of Hail, Hail 55471, Saudi Arabia; h.bamahros@uoh.edu.sa; 4 Department of Medicine, Division of Endocrinology, Diabetes, and Metabolism, College of Medicine, University of Illinois Chicago, Chicago, IL 60612, USA; amahmo4@uic.edu; 5 Department of Kinesiology and Nutrition, College of Applied Health Sciences, University of Illinois at Chicago, Chicago, IL 60607, USA; 6 Centre International de Recherche en Infectiologie, CIRI, Inserm, U1111, 69007 Lyon, France; emily.sible@ens-lyon.fr (E.S.); uzma.hasan@inserm.fr (U.A.H.); 7 Cancer Research Centre of Lyon, CRCL, INSERM U1052-CNRS UMR5286, 69008 Lyon, France

**Keywords:** breast cancer, Toll-like receptor 9, systematic review

## Abstract

**Simple Summary:**

Breast cancer is a major global issue, ranking as the second most common cancer among women. Various factors such as genetics, epigenetics, obesity, sedentary lifestyle, tobacco use, and vitamin D deficiency have been implicated in its prevalence. Toll-like receptor 9 (TLR9) has emerged as a potential key factor, yet its role in breast cancer has not been fully explored. TLR9, a component of the innate immune system, recognizes unmethylated CpG motifs, thus initiating immune responses. Understanding TLR9 expression and function in breast tissue, particularly in distinguishing between normal and cancerous tissue, is crucial. This review examines the existing literature on TLR9 expression in both normal and cancerous breast tissue, aiming to identify knowledge gaps and enhance our understanding of TLR9’s role in breast cancer. The ultimate goal is to enhance our understanding of TLR9’s involvement in breast cancer pathogenesis, which could potentially lead to novel diagnostic markers or therapeutic targets, thereby improving patient outcomes and advancing breast cancer treatment strategies.

**Abstract:**

Breast cancer is a significant global issue, ranking as the second most common cancer among women worldwide and a leading cause of cancer-related deaths. Although the exact causes of this increase remain unclear, factors such as genetics, epigenetics, obesity, sedentary lifestyle, tobacco use, and vitamin D deficiency have been implicated. The Toll-like receptor 9 (TLR9) is recognized for its role in inflammation and innate immunity; however, its specific involvement in breast cancer pathogenesis requires further investigation. This study aims to systematically review the existing literature on TLR9 expression in normal and cancerous breast tissue, providing current knowledge and identifying gaps. Relevant articles in English were from PubMed, Scopus, and Google Scholar, with the inclusion criteria focusing on studies evaluating TLR9 mRNA and protein expression. The review found that TLR9 mRNA and protein exhibit variable expressions in both normal and cancerous breast tissue, highlighting the need for further research to clarify TLR9’s role in breast cancer.

## 1. Introduction

Breast cancer, the most prevalent cancer diagnosed in women worldwide, remains one of the leading causes of cancer-related deaths. According to IARC Globocan 2022, approximately 2.3 million women were diagnosed with breast cancer, and about 670,000 died in 2022, with considerable geographic variations in burden across countries and world regions [1]. Forecasts suggest that by 2040, the burden of breast cancer could increase to more than 3 million new cases and 1 million deaths annually, mainly due to population growth and aging [2].

Breast cancer is a clinically and biologically heterogeneous disease that evolves through genetic, epigenetic, and modifiable environmental factors, which influence its initiation and progression [3,4]. Originating in the female breast, which encompasses a network of lobules, ducts, and stroma, breast cancer emerges as a transformation of normal epithelial cells. Typically, breast cancer develops in situ and presents predominantly as ductal carcinoma. Cancerous cells invade the basement membrane and metastasize to distant organs as the disease progresses. Although diagnosis and treatments have advanced, improving outcomes and life quality for women battling breast cancer, the alarming statistic that 9% of these patients succumb to the disease underscores the crucial need for novel therapeutic approaches and the identification of new targets [5]. Recently, there has been growing interest in the role of Toll-like receptors in cancer, including breast cancer. In the following sections, we will summarize their ability to modulate the tumor microenvironment, influence tumor growth, and affect the immune response against tumors.

**Figure 1:**
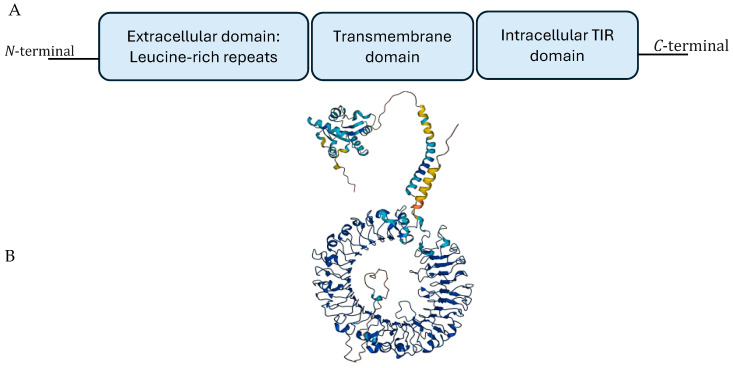
(**A**) Schematic diagram of TLR9 domains and (**B**) three-dimension crystal structure of unliganded TLR9.

Toll-like receptors (TLRs) constitute a class of evolutionarily conserved pattern-recognition receptors. Ten TLRs have been identified in humans, and twelve in mice, that are grouped by their cellular location: TLRs on the cell surface include TLR1, TLR2, TLR4, TLR5, TLR6, and TLR11, which mainly recognize membrane components such as lipids, lipoproteins, and proteins on bacteria, while intracellular TLRs include TLR3, TLR7, TLR8, and TLR9, which recognize viral and bacterial nucleic acids [6,7]. TLRs are expressed in immune and non-immune cells, playing a crucial role in the innate immune system by identifying pathogens through the detection of pathogen-associated molecular patterns (PAMPs) originating from various sources such as bacteria, viruses, fungi, protozoa, and damage-associated molecular patterns (DAMPs) from injured tissues [8]. TLRs belong to type I transmembrane glycoproteins and comprise distinct structural elements, including an extracellular domain responsible for the binding of PAMPs, characterized by leucine-rich repeat motifs, a single transmembrane domain with a single α-helix, and an intracellular Toll-IL-1 receptor (TIR) domain, which serves as an initiator of signaling cascades [9]. The three-dimensional (3D) structure of TLR9 in Figure 1 was obtained from gene cards [10]. This recognition activates downstream-signaling cascades to secrete cytokines and chemokines, triggering an immune response that engages innate and adaptive components [11]. However, aberrant activation of TLRs has also been linked to the pathogenesis of an increasing number of diseases [12,13,14,15,16].

The role of TLRs in breast cancer is controversial. Some studies, as cited in the following section, reported pro-tumor effects of certain TLRs on breast cancer cells, promoting tumor growth and metastasis. For instance, TLR4 activation has been linked to increased cell proliferation, invasion, and resistance to apoptosis. It can also promote an inflammatory environment that supports tumor growth [17]. Conversely, activation of other TLRs can induce anti-tumor immune responses. For example, TLR3 activation can trigger apoptosis in breast cancer cells and enhance the immune system’s ability to recognize and destroy cancer cells [18]. TLR activation can produce pro-inflammatory cytokines and chemokines that recruit immune cells to the tumor site. This can have a dual effect; while inflammation can sometimes promote tumor growth, it can also enhance the recruitment and activation of cytotoxic immune cells that target the tumor [14]. Some TLRs can also contribute to an immunosuppressive tumor microenvironment. For example, TLR2 and TLR4 activation may induce regulatory T cells (Tregs) and myeloid-derived suppressor cells (MDSCs), which inhibit anti-tumor immune responses [19]. Therefore, understanding the complex roles of different TLRs in breast cancer is crucial. This review highlights TLR9, one of the TLRs linked to breast cancer growth and response to therapy.

**Figure 2:**
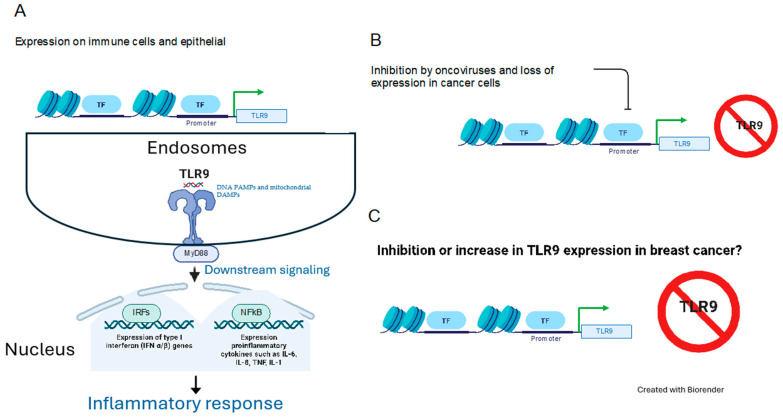
TLR9 regulation in cancer. (**A**) TLR9 promoter expression is autoregulated in immune and non-immune cells, leading to downstream signaling and the induction of an inflammatory response. (**B**) Several oncoviruses downregulate TLR9 protein expression by promoter inhibition. (**C**) However, TLR9 expression (inhibited or increased) and mode of regulation in breast cancer remains a topic of ongoing debate. Abbreviations: TF: transcription factors (TF).

TLR9 is an intracellular sentinel that recognizes unmethylated DNA PAMPs [8] and mitochondrial DAMPs [20]. Situated on human chromosome 3 (3p21.3), the *TLR9* gene has five isoforms generated by alternative splicing with a cell-specific expression of *TLR9* isoforms during inflammation [21]. Synthesized within the endoplasmic reticulum, TLR9 is then transported to endosomes, which transmit downstream signals (Figure 2). The signal transduction pathway mediated by TLR9, which is common to other members of the TLR family, involves several key molecules: myeloid differentiation primary response 88 (MyD88), IL-1 receptor-associated kinase (IRAK), TNF receptor-associated factor 6 (TRAF6), TGFβ-activated kinase 1 (TAK1), IκB kinases, IκB, and NF-κB [22,23]. Its expression is not confined solely to immune cells, such as monocytes, macrophages, plasmacytoid dendritic cells, and T and B lymphocytes; it also extends to nonimmune cells, such as epithelial cells [24,25] and neuronal system [26]. Activated TLR9 has been shown to play a role in the invasion and metastasis of cancer cells in vitro [24,27,28]. Synthetic ligands targeting TLR9, mimicking the structure of bacterial DNA, have been shown to induce cancer cell invasion in vitro, suggesting a potential link between TLR9 activation and cancer progression [29]. Oxidative stress has been identified as a regulator of TLR expression, indicating a possible mechanism through which environmental factors influence TLR-mediated responses [30]. Their involvement has also been implicated in the pathogenesis of autoimmune diseases, neurocognitive deficiency, obesity-related metabolic disorders, and cardiometabolic disorders [12,26,31,32,33], underscoring their multifaceted role in both health and disease.

Mounting evidence underscores the significant link between TLR9 and the occurrence and development of breast cancer. Therefore, targeting these receptors may be a potential strategy for treating breast cancer. TLR9 signaling may regulate breast cancer cell growth, metastasis, and apoptosis. Notably, the administration of a tumor-targeting TLR9 agonist, PIP-CpG, induced a systemic T cell-mediated immune response, promoting the regression of existing mammary tumors and eliciting an immune memory response that delayed the growth of new tumors [34]. The expression of Toll-like receptors in cancer cells is linked to tumor proliferation, survival, and the ability to manipulate the tumor microenvironment to support tumorigenesis. The role of TLR9 in cancer development remains controversial, with antitumor and pro-tumor characteristics reported in the literature. This review systematically analyzes studies investigating TLR9 expression in normal and tumor breast tissue.

## 2. Methods and Results

### 2.1. Literature Search and Study Selection

There is a crucial need to establish a consensus on the level of TLR9 in breast cancer tissue, as it could be considered a potential predictive marker for breast cancer. A literature review of TLR9 expression was conducted using the Medical Subject Headings (MeSH) function on pubmed.ncbi.nlm.nih.gov (last accessed on 14 May 2024). A search was also performed using the search terms “Toll-like receptor 9” [Mesh] AND “Breast Neoplasms” [Mesh]. Scopus and Google Scholar were searched, using “TLR9” and “breast cancer” as the keywords in the titles. The Wiley database was searched using “Toll-like receptor 9” anywhere, “breast cancer” in the title, and “TLR9” anywhere. And the Embase database was searched with Query ’breast cancer’:ti AND ‘toll like receptor 9’:ti. All references were screened for eligibility by evaluating publication titles and abstracts. Each article was then fully assessed in accordance with the eligibility criteria described below.

### 2.2. Inclusion and Exclusion Criteria

**Figure 3:**
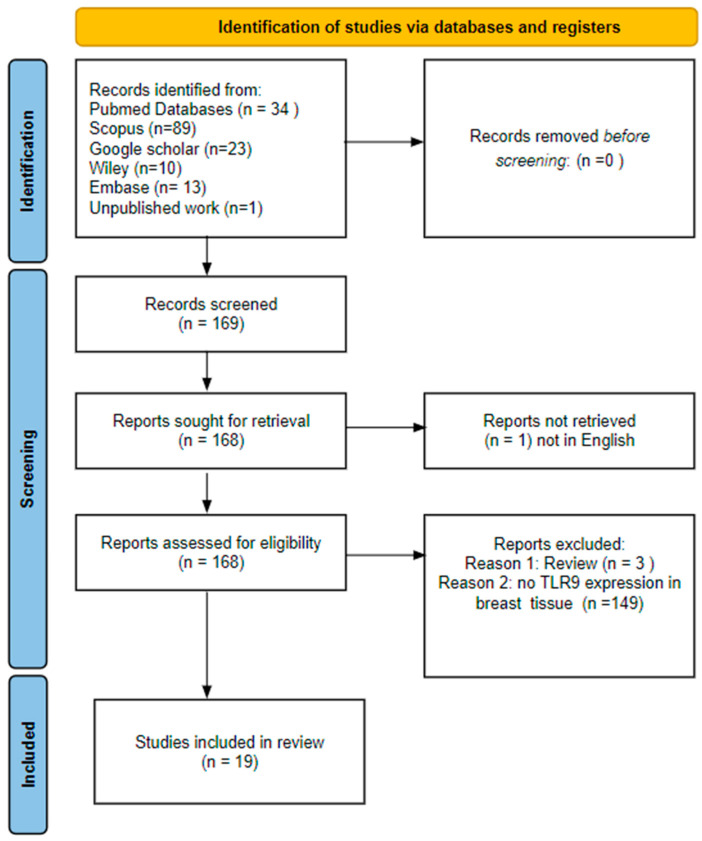
Prisma flow diagram of the literature search results for the systematic review.

The inclusion criteria focused on articles relevant to breast neoplasms and TLR9 that reported human TLR9 expression results (mRNA, Western blot, immunohistochemistry, or ELISA). Additional criteria required articles to be accessible and available in English. Exclusion criteria eliminated articles not written in English, those assessing other diseases, or those not reporting TLR9 expression (protein or mRNA). The search and evaluation processes are summarized in Figure 3 and yielded 19 results. Two of the authors independently reviewed the databases, and disagreements were resolved by consensus.

### 2.3. Data Extraction and Analysis

Table 1 provides a comprehensive overview of the findings of the literature review on TLR9 expression in breast cancer. A total of 19 studies were analyzed, revealing a widespread expression of TLR9 in breast tissue. The following data were extracted from all the studies: type of breast tissue sample, total number of cases, TLR9 detection method, and summary of findings. Most studies were case–control or case-only studies using archival breast tissue or serum to assess the correlation between TLR9 expression and breast cancer clinicopathological characteristics. The source population included participants from Europe, the USA, China, the Middle East, and India, encompassing various subtypes and progressions of breast cancer. Antibodies from Imgenex were commonly used for IHC. The systematic review identified heterogeneous relationships between TLR9 and breast cancer, highlighting the complexity of its involvement in the disease.

### 2.4. TLR9 Expression and Cellular Localization in Breast Tissue

Several studies have confirmed the TLR9 mRNA and protein’s variable expression in normal and cancerous breast tissue, as well as breast cell lines. Amongst the five human TLR9 isoforms, the expression of the TLR9 A and B isoforms has been detected in breast tissue [35]. There is a correlation between the TLR9 mRNA and protein expression levels, indicating that any TLR9 deregulation may be mainly transcriptional in breast tumors [36]. Merrell et al., using a small sample size, observed that the TLR9 protein was expressed in normal and breast cancer tissue; they also noted that the TLR9 Western blot band detected in normal mammary gland tissue appeared slightly heavier than the TLR9 band in the malignant tumors, indicating a higher level of TLR9 [37]. When TLR9 protein expression was visualized in breast tissues via immunohistochemistry (IHC), TLR9 staining was localized in the cytoplasm, in particular, the endocytic compartment of the epithelial cells in both cancer and normal samples, and not in stroma cells [29,38]. However, other studies reported cytoplasmic TLR9 staining in the tumor and adjacent stroma [36,39]. The carcinoma cells exhibited diffuse cytoplasmic TLR9 staining with varying intensities, contrasting with the normal epithelial cells, where TLR9 was localized only in the apical part, facing the lumen of the breast ducts. The TLR9 protein was found to be localized in malignant epithelial cancer cells and adjacent stromal cells, mainly stromal myofibroblast and mononuclear inflammatory cells [40]. Although there are reports that surface TLR9 (sTLR9) may be expressed in immune cells and breast cancer cell lines, the contribution of this expression to the growth or survival of either normal or tumorigenic breast cells is completely undefined at present [41].

### 2.5. Upregulation of TLR9 and Association with Breast Clinicopathological Characteristics of Breast

Several studies have shown that TLR9 is upregulated in breast cancer tissue and serum, which could potentially participate in promoting breast cancer development and metastasis [27,29,36,38,39,42,43,44,45,46]. The overexpression of TLR9 in breast cancer tissue was associated with both adverse and favorable outcomes.

For example, Ilvesaro et al. demonstrated the epithelial localization of TLR9 in both cancerous and non-cancerous samples, with a significantly higher stain intensity in cancer cells than in normal cells [29]. Jukkola-Vuorinen et al. found a widespread expression of TLR9 in tumors [38]. They observed an inverse relationship between TLR9 and ER expression levels, and the TLR9 expression correlated with high-grade tumors and specific histological subtypes. Berger et al. observed the same inverse relationship between ER and TLR9 (mRNA and/or protein), which correlated with high-grade and NF-κB activity [42]. Meseure et al. observed that TLR9 overexpression was associated with changes in downstream components of the TLR9-signaling pathway, epithelial–mesenchymal transition (EMT) induction, and deregulation of the epidermal growth factor receptor pathways [36]. Similarly, Qiu et al. linked high TLR9 expression to an advanced pathological stage, larger tumor size, estrogen receptor (ER) negativity, and positive lymph node metastasis in breast cancer [27]. ÇElİK et al. found an association between TLR9 up-regulation and high-grade, triple-negative, and low overall survival [44]. A correlation was observed between lymph node metastasis and high TLR9 expression in malignant epithelial cells [40].

Interestingly, low tumor TLR9 levels have been associated with poor survival in Caucasian patients with triple-negative breast cancer (TNBC) [47]. Conversely, a high TLR9 protein expression did not protect against relapse in African American TNBC patients. Chandler et al. observed a lower frequency of a protective TLR9 variant in African American breast cancer patients, highlighting racial disparities in TLR9-associated protection against relapse [48]. González-Reyes et al. observed a significant increase in TLR9 mRNA levels in recurrent carcinomas, particularly in fibroblast-like cells, highlighting its association with metastatic potential [43]. Additionally, TLR9 levels were found to be elevated in the serum of breast cancer patients compared to normal patients, as measured by an enzyme-linked immunosorbent assay (ELISA) [45,49]. Moaz et al. also found that higher circulating TLR9 levels correlated with metastasis [45].

Although the studies above established a correlation between TLR9 and cancer progression or adverse outcomes, TLR9 overexpression was linked to favorable results in patients receiving neoadjuvant chemotherapy. Singh et al. correlated high TLR9 expression with improved overall survival in breast cancer patients receiving neoadjuvant chemotherapy, suggesting that high TLR9 expression may indicate the initiation of antitumor activity by DNA fragments from chemotherapy-treated dead cancer cells, with a significant association with age or histopathological stage [39].

### 2.6. Downregulation of TLR9 and Association with Breast Clinicopathological Characteristics

In contrast, several studies have observed the downregulation of TLR9 in breast cancer, indicating its potential role as a tumor suppressor [30,46,47,50,51]. This suggests that the modulation of TLR9 expression is necessary for tumor progression. Shi et al. observed a decrease in TLR9 mRNA among 1215 patients with breast cancer in the Cancer Genome Atlas (TCGA) database compared to matched normal tissues; this decrease was correlated with those of interferon-β and C-X-C motif chemokine ligand 10 [51]. However, Shi et al. also demonstrated that a high level of TLR9 was strongly associated with ER-negative subtypes (HER2-enriched and triple-negative) [51]. Karki et al. observed lower TLR9 serum levels with advanced clinical characteristics in patients with malignant breast cancer [30,50]. Tuomela et al. associated low tumor TLR9 expression with shorter disease-specific survival in patients with triple-negative breast cancer [47]. Recently, our group observed a reduction in TLR9 protein expression in tumor tissue compared to adjacent and normal breast tissue [46].

**Table 1:** Summary of the expression of the TLR9 in breast tissue and serum.

Author	Breast Samples	Total Cases	TLR9 Detection Method	TLR9 Expression
Merrell [37]	Lysate	6	WB: Img-431, Imgenex, Bhubaneswar, Odisha, India	Expressed in normal and tumor
Sandholm [35]	FFPE	196	mRNA	Downregulated in ER-positive
Singh A [40]	FFPE	38	IHC: PA5-20202, Thermo Fischer, Waltham, MA, USA	TLR9 in epithelial and stromal
**Upregulation of TLR9 in breast cancer**				
Ilvesaro [29]	FFPE	35	IHC: Img-305A, Imgenex	Upregulated in tumor
Jukkola-Vuorinen [38]	FFPE	141	IHC: Img-305A, Imgenex	Upregulated in aggressive tumor
Berger [42]	FFPE and Frozen	240	WB: sc13218, Santa Cruz, Santa Cruz, CA, USA	Upregulated in aggressive tumors
González-Reyes [43]	FFPE	74	IHC &WB: sc-25468, Santa Cruz	Upregulated in aggressive tumor
Qiu [27]	FFPE	124	IHC: 2254, Cell signaling, Danvers, MA, USA	Upregulated in aggressive tumor
Meseure [36]	FFPE and Frozen	480	IHC: Img-305A, Imgenex	Upregulated in tumor
Chandler [48]	FFPE	51	IHC: Img-305A; Imgenex	Upregulated in triple-negative
Abdulwahid [49]	Serum	120	ELISA (Elabscience, Houston, TX, USA)	Upregulated in tumor
Singh [39]	FFPE	42	IHC: PA5-20202, Thermo Fischer	Upregulated in tumor
ÇElİK [44]	FFPE	139	IHC: ab37154, Abcam, Cambridge, UK	Upregulated in aggressive tumor
Moaz [45]	Serum	186	ELISA: SG11478, Sino Gene Clone Biotech, Beijing, China	Upregulated in aggressive tumor
**Downregulation of TLR9 in breast cancer**				
Tuomela [47]	FFPE	12	WB: Img-431, Imgenex	Downregulated in triple-negative
Karki [50]	Serum	180	ELISA: USCN Life Science & Technology, Wuhan, China	Downregulated in tumor
Karki [30]	Serum	210	ELISA: USCN Life Science & Technology	Downregulated in tumor
Shi [51]	TCGA	1215	mRNA	Downregulated in tumor
Sible [46]	FFPE	244	IHC: Cell Signaling	Downregulated in tumor

Abbreviations: FFPE: formalin-fixed paraffin-embedded breast tissue, IHC: immunohistochemical staining, WB: Western blot, TCGA: The Cancer Genome Atlas.

## 3. Discussion

Toll-like receptors (TLRs) are pivotal players in both innate and adaptive immune responses, orchestrating defenses against microbial invasion and tissue damage. Beyond their traditional role in immune cells, growing evidence suggests their involvement in cancer biology, where they influence carcinogenesis and tumor progression, particularly within the inflammatory milieu of the tumor microenvironment [52].

**Figure 4:**
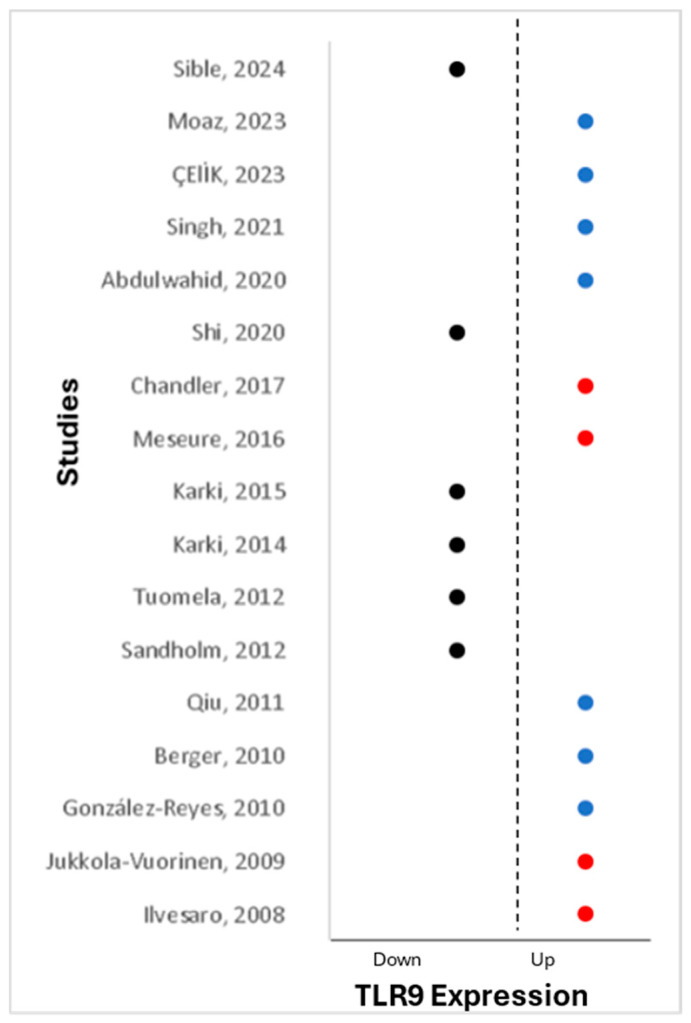
A graphic display of the discrepancy of TLR9 expression across the selected studies organized by their year of publication. Black dots are studies that reported the down regulation of TLR9. Blue dots are the studies that reported the upregulated TLR9. The red dots represent the studies that used the same antibody (Img,305A, Imgenex, 11175 Flintkote Avenue Suite E San Diego, CA 92121 United States) to detect TLR9 [27,29,30,35,36,38,39,42,43,44,45,46,47,48,49,50,51].

TLR9 stands out among innate immune receptors as the first identified receptor recognizing double-stranded DNA (dsDNA), activated by specific unmethylated CpG motifs in viral genomes [8]. Alongside other innate receptors, TLR9, expressed by plasmacytoid dendritic cells, macrophages, B cells, and T-cells, produces type I interferon (IFN) and pro-inflammatory cytokines that are crucial for controlling viral infections and enhancing innate and adaptive memory responses. Differential expressions of TLR9 have been observed between normal and tumor cells in various cancers, including head and neck cancer [53], B-cell chronic lymphocytic leukemia [13], colorectal cancer [54], and glioma [28]. Furthermore, TLR9 polymorphisms are implicated in breast cancer susceptibility [55]. The systematic review identified heterogeneous relationships between TLR9 and breast cancer, highlighting the complexity of its involvement in the disease. TLR9 has been shown to be expressed in normal and cancerous breast tissue, but its association with breast cancer development and progression is inconsistent. A meta-analysis was not performed as effect estimates were not reported. Instead, we present a graphical representation of TLR9 expression (both protein and mRNA) across various studies, organized by their year of publication (Figure 4). The red dots in Figure 4 highlight the studies that utilized the same antibody (Img,305A, Imgenex) to detect TLR9. Understanding the dual role of TLR9 in breast cancer—both as a guardian against tumor cells and as a facilitator of tumor progression—is crucial. Further research is needed to unravel the intricacies of TLR9’s function in breast cancer and its prognostic implications, paving the way for targeted therapeutic interventions.

### 3.1. Discrepancies Cellular and Subcellular Sublocalization of TLR9 in Breast Tissue

**Figure 5:**
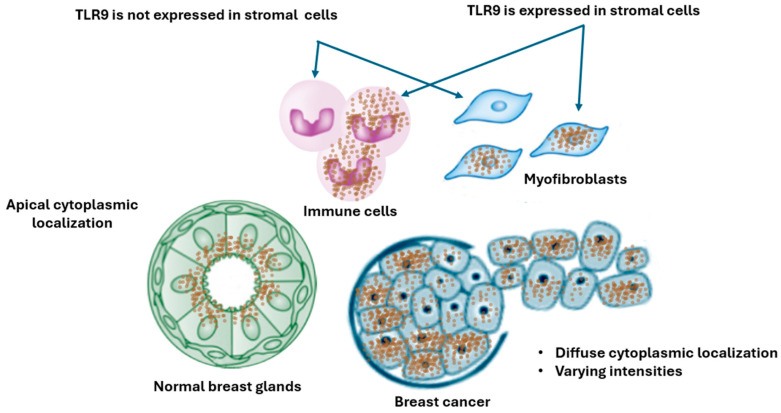
TLR9 expression in normal and tumor breast tissue.

Our results highlight the need to better understand the cellular and subcellular localization of TLR9 in both normal and tumor breast tissue. TLR9 is expressed in various cell types within breast tissue, including epithelial cells, plasmacytoid dendritic cells, macrophages, and B cells, significantly shaping immune surveillance and response. TLR9 is localized within endosomes, where it recognizes and binds to unmethylated CpG DNA, initiating downstream-signaling pathways that lead to immune activation [52]. Discrepancies in TLR9 expression levels between normal and cancerous breast tissue were observed, which can arise due to several factors. Detection techniques such as mRNA analysis, Western blotting, and immunohistochemical staining yield varying results. The standardization of these methods is essential for consistent and comparable data. The immunohistochemical staining revealed diffuse cytoplasmic TLR9 staining in the epithelial cells of both cancerous and normal breast tissue samples. However, the intensity, frequency, and type of cells expressing TLR9 varied across different studies. The choice between whole-slide and tissue microarray can affect the ability to visualize heterogeneity within the examined tissue. The specificity and sensitivity of antibodies to detect TLR9 can significantly affect the results. High-quality, validated antibodies are crucial for accurate localization studies. A comprehensive study involving 1215 patients with breast cancer from the TCGA database revealed that the expression of TLR9 mRNA was generally lower in breast cancer cases compared to normal tissue; however, it was found to be higher in the estrogen-receptor/progesterone-receptor-negative breast cancer cases compared to the control tissue [51]. It is important to note that mRNA analysis does not identify the specific cells expressing TLR9. The variability of TLR9 expression across several studies can be attributed to differential expression in distinct cell types, highlighting the necessity of identifying the specific cells expressing TLR9. Figure 5 provides a summary of TLR9 expression in normal and tumor breast tissue, as reported in the studies included in this systematic review. This identification can be achieved through multicolor immunohistochemical staining, which allows for clear visualization of TLR9-expressing cells. Additionally, spatial gene expression and single-cell RNA sequencing (scRNA-seq) techniques can be employed to examine TLR9 expression within specific cell types. These methods will clarify the precise localization and expression patterns of TLR9 in breast tissue, providing deeper insights into its role in breast cancer. Variability in patient samples, including differences in sample size, demographic factors, and clinical characteristics, can lead to inconsistent findings. To draw robust conclusions, larger and well-characterized cohorts are needed.

### 3.2. Dysregulation of TLR9 and Association with Breast Clinicopathological Characteristics

The upregulation of TLR9 in breast cancer has been associated with various clinicopathological characteristics, including more aggressive tumor features, such as higher grade, larger size, and increased lymph node involvement. Furthermore, the upregulation of TLR9 has been linked to specific breast cancer subtypes, particularly estrogen-receptor-negative (ER-) and triple-negative breast cancers (TNBC), which are known to exhibit a poorer prognosis and limited treatment options compared to hormone-receptor-positive tumors. This association underscores the clinical relevance of TLR9 expression in the definition of breast cancer subtypes and the guidance of personalized treatment strategies. Furthermore, studies have suggested a potential role for TLR9 upregulation in modulating the tumor microenvironment and immune response. Elevated TLR9 expression can enhance the inflammatory response within the tumor microenvironment, promoting tumor growth and progression. Additionally, TLR9 upregulation has been implicated in immune evasion mechanisms employed by breast cancer cells, leading to resistance to specific therapeutic interventions and poorer clinical outcomes.

Conversely, the downregulation of TLR9 in breast cancer has also been reported in some studies. Sandholm et al. have detected a decrease in TLR9 expression in ER+ breast cancer cell lines regulated by Erα [35]. Furthermore, the stimulation of TLR9-expressing breast cancer cells with the TLR9 agonist CpG oligonucleotides increased their invasion in vitro [37]. Finally, specific TLR9 polymorphisms have been implicated in breast cancer susceptibility, suggesting a genetic influence on TLR9 expression and its role in cancer development.

TLR9 plays a complex role in breast cancer, acting both as a protector and a promoter of tumor development. This duality raises the key question: what factors dictate TLR9’s divergent roles in breast cancer? TLR9 promoter expression is autoregulated in both immune and non-immune cells [23], and its expression is known to be downregulated by several oncoviruses [56,57,58,59]. On one hand, TLR9 can combat breast cancer cells by recognizing DAMPs and triggering type 1 immune responses to eliminate tumor cells. On the other hand, TLR9 upregulation may facilitate tumor progression and metastasis. The prognostic significance of TLR9 in breast cancer remains an area that requires further exploration. Regulation of TLR9 expression in breast cancer is complex and context-dependent.

## 4. Conclusions

In summary, TLR9 is emerging as a crucial determinant of breast cancer clinicopathological characteristics, including tumor aggressiveness, response to treatment, and immune evasion. Regulation of TLR9 within breast cancer is complex, and studies report both upregulation and downregulation. These conflicting findings emphasize the pressing need for further investigations to delineate the cellular and subcellular localization of TLR9 in breast tissue, its precise role in breast cancer pathogenesis, and its potential as a therapeutic target. The involvement of TLR9 in promoting inflammation and enhancing invasive capacities highlights the intricate interplay between the immune system and cancer progression. Currently, therapeutic strategies lack a comprehensive understanding of the immune networks that govern breast cancer development, requiring the exploration of innovative approaches. Beyond its implications in cancer, TLR9 is significant in adipose tissue biology, insulin resistance, and oxidative stress. Its potential as a prognostic marker and therapeutic target underscores its clinical relevance. A comprehensive exploration of the molecular mechanisms that underlie TLR9 dysregulation in breast cancer and its functional implications is imperative. Such efforts promise to shed light on their role in breast cancer development and progression and offer avenues to identify novel therapeutic targets, ultimately enhancing clinical outcomes for patients with this heterogeneous disease.

## Data Availability

The data supporting the findings of this study are available within the article.

## References

[1] Ferlay J., Ervik M., Lam F., Laversanne M., Colombet M., Mery L., Piñeros M., Znaor A., Soerjomataram I., Bray F. Global Cancer Observatory: Cancer Today. https://gco.iarc.who.int/today.

[2] Arnold M., Morgan E., Rumgay H., Mafra A., Singh D., Laversanne M., Vignat J., Gralow J.R., Cardoso F., Siesling S. (2022). Current and future burden of breast cancer: Global statistics for 2020 and 2040. Breast.

[3] Place A.E., Jin Huh S., Polyak K. (2011). The microenvironment in breast cancer progression: Biology and implications for treatment. Breast Cancer Res..

[4] Guidroz J.A., Weigel R.J., Dirbas F., Scott-Conner C. (2011). The Biology of Breast Cancer. Breast Surgical Techniques and Interdisciplinary Management.

[5] American Cancer Society Breast Cancer Facts & Stats 2024—Incidence, Age, Survival, & More. https://www.cancer.org/cancer/types/breast-cancer/about/how-common-is-breast-cancer.html.

[6] O’Neill L.A.J., Golenbock D., Bowie A.G. (2013). The history of Toll-like receptors—Redefining innate immunity. Nat. Rev. Immunol..

[7] Weiss H.J., O’Neill L.A.J. (2022). Of Flies and Men-The Discovery of TLRs. Cells.

[8] Hemmi H., Takeuchi O., Kawai T., Kaisho T., Sato S., Sanjo H., Matsumoto M., Hoshino K., Wagner H., Takeda K. (2000). A Toll-like receptor recognizes bacterial DNA. Nature.

[9] Gay N.J., Gangloff M., Weber A.N. (2006). Toll-like receptors as molecular switches. Nat. Rev. Immunol..

[10] Genecards TLR9 Gene—Toll Like Receptor 9. https://www.genecards.org/cgi-bin/carddisp.pl?gene=TLR9#proteins-structures.

[11] Kawasaki T., Kawai T. (2014). Toll-like receptor signaling pathways. Front. Immunol..

[12] Means T.K., Latz E., Hayashi F., Murali M.R., Golenbock D.T., Luster A.D. (2005). Human lupus autoantibody-DNA complexes activate DCs through cooperation of CD32 and TLR9. J. Clin. Investig..

[13] Rybka J., Butrym A., Wróbel T., Jaźwiec B., Bogucka-Fedorczuk A., Poręba R., Kuliczkowski K. (2016). The Expression of Toll-like Receptors in Patients with B-Cell Chronic Lymphocytic Leukemia. Arch. Immunol. Ther. Exp..

[14] Cen X., Liu S., Cheng K. (2018). The Role of Toll-Like Receptor in Inflammation and Tumor Immunity. Front. Pharmacol..

[15] Nokhandani N., Naghavi-Alhosseini M., Davoodi H. (2019). The role of toll-like receptors in breast cancer. J. Inflamm. Dis..

[16] Davakis S., Kapelouzou A., Liakakos T., Mpoura M., Stergiou D., Sakellariou S., Charalabopoulos A. (2022). The Role of Toll-like Receptors in Esophageal Cancer. Anticancer Res..

[17] Ahmed A., Redmond H.P., Wang J.H. (2013). Links between Toll-like receptor 4 and breast cancer. Oncoimmunology.

[18] Salaun B., Coste I., Rissoan M.C., Lebecque S.J., Renno T. (2006). TLR3 can directly trigger apoptosis in human cancer cells. J. Immunol..

[19] Zhou H., Jiang M., Yuan H., Ni W., Tai G. (2021). Dual roles of myeloid-derived suppressor cells induced by Toll-like receptor signaling in cancer. Oncol. Lett..

[20] Zhang Q., Raoof M., Chen Y., Sumi Y., Sursal T., Junger W., Brohi K., Itagaki K., Hauser C.J. (2010). Circulating mitochondrial DAMPs cause inflammatory responses to injury. Nature.

[21] McKelvey K.J., Highton J., Hessian P.A. (2011). Cell-specific expression of TLR9 isoforms in inflammation. J. Autoimmun..

[22] Kawai T., Sato S., Ishii K.J., Coban C., Hemmi H., Yamamoto M., Terai K., Matsuda M., Inoue J., Uematsu S. (2004). Interferon-α induction through Toll-like receptors involves a direct interaction of IRF7 with MyD88 and TRAF6. Nat. Immunol..

[23] Takeshita F., Suzuki K., Sasaki S., Ishii N., Klinman D.M., Ishii K.J. (2004). Transcriptional regulation of the human TLR9 gene. J. Immunol..

[24] Sandholm J., Selander K.S. (2014). Toll-like receptor 9 in breast cancer. Front. Immunol..

[25] Alzahrani B. (2020). The Biology of Toll-Like Receptor 9 and Its Role in Cancer. Crit. Rev. Eukaryot. Gene Expr..

[26] Jovasevic V., Wood E.M., Cicvaric A., Zhang H., Petrovic Z., Carboncino A., Parker K.K., Bassett T.E., Moltesen M., Yamawaki N. (2024). Formation of memory assemblies through the DNA-sensing TLR9 pathway. Nature.

[27] Qiu J., Shao S., Yang G., Shen Z., Zhang Y. (2011). Association of Toll like receptor 9 expression with lymph node metastasis in human breast cancer. Neoplasma.

[28] Fehri E., Ennaifer E., Bel Haj Rhouma R., Ardhaoui M., Boubaker S. (2022). TLR9 and Glioma: Friends or Foes?. Cells.

[29] Ilvesaro J.M., Merrell M.A., Li L., Wakchoure S., Graves D., Brooks S., Rahko E., Jukkola-Vuorinen A., Vuopala K.S., Harris K.W. (2008). Toll-like receptor 9 mediates CpG oligonucleotide-induced cellular invasion. Mol. Cancer Res. MCR.

[30] Karki K., Pande D., Negi R., Khanna S., Khanna R.S., Khanna H.D. (2015). Correlation of serum toll like receptor 9 and trace elements with lipid peroxidation in the patients of breast diseases. J. Trace Elem. Med. Biol..

[31] Hong C.-P., Yun C.H., Lee G.-W., Park A., Kim Y.-M., Jang M.H. (2015). TLR9 regulates adipose tissue inflammation and obesity-related metabolic disorders. Obesity.

[32] Nishimoto S., Fukuda D., Sata M. (2020). Emerging roles of Toll-like receptor 9 in cardiometabolic disorders. Inflamm. Regen..

[33] Zhang Y., Liu J., Wang C., Liu J., Lu W. (2021). Toll-Like Receptors Gene Polymorphisms in Autoimmune Disease. Front. Immunol..

[34] Miller C.L., Sagiv-Barfi I., Neuhöfer P., Czerwinski D.K., Bertozzi C.R., Cochran J.R., Levy R. (2023). Targeted TLR9 Agonist Elicits Effective Antitumor Immunity against Spontaneously Arising Breast Tumors. J. Immunol..

[35] Sandholm J., Kauppila J.H., Pressey C., Tuomela J., Jukkola-Vuorinen A., Vaarala M., Johnson M.R., Harris K.W., Selander K.S. (2012). Estrogen receptor-α and sex steroid hormones regulate Toll-like receptor-9 expression and invasive function in human breast cancer cells. Breast Cancer Res. Treat..

[36] Meseure D., Vacher S., Drak Alsibai K., Trassard M., Nicolas A., Leclere R., Lerebours F., Guinebretiere J.M., Marangoni E., Lidereau R. (2016). Biopathological Significance of TLR9 Expression in Cancer Cells and Tumor Microenvironment Across Invasive Breast Carcinomas Subtypes. Cancer Microenviron..

[37] Merrell M.A., Ilvesaro J.M., Lehtonen N., Sorsa T., Gehrs B., Rosenthal E., Chen D., Shackley B., Harris K.W., Selander K.S. (2006). Toll-like receptor 9 agonists promote cellular invasion by increasing matrix metalloproteinase activity. Mol. Cancer Res..

[38] Jukkola-Vuorinen A., Rahko E., Vuopala K.S., Desmond R., Lehenkari P.P., Harris K.W., Selander K.S. (2009). Toll-like receptor-9 expression is inversely correlated with estrogen receptor status in breast cancer. J. Innate Immun..

[39] Singh A., Bandyopadhyay A., Mukherjee N., Basu A. (2021). Toll-Like Receptor 9 Expression Levels in Breast Carcinoma Correlate with Improved Overall Survival in Patients Treated with Neoadjuvant Chemotherapy and Could Serve as a Prognostic Marker. Genet. Test. Mol. Biomark..

[40] Singh A., Bandyopadhyay A., Mukherjee N., Basu A. (2020). α-Smooth Muscle Actin and TLR9 Expression and Correlation in Breast Cancer. Int. J. Pathol. Clin. Res..

[41] Kou M., Wang L. (2023). Surface toll-like receptor 9 on immune cells and its immunomodulatory effect. Front. Immunol..

[42] Berger R., Fiegl H., Goebel G., Obexer P., Ausserlechner M., Doppler W., Hauser-Kronberger C., Reitsamer R., Egle D., Reimer D. (2010). Toll-like receptor 9 expression in breast and ovarian cancer is associated with poorly differentiated tumors. Cancer Sci..

[43] González-Reyes S., Marín L., González L., González L.O., del Casar J.M., Lamelas M.L., González-Quintana J.M., Vizoso F.J. (2010). Study of TLR3, TLR4 and TLR9 in breast carcinomas and their association with metastasis. BMC Cancer.

[44] ÇElİK Z.E., DemİR F., Yonar H., ÇElİK M., Eren O.Ö. (2023). Association of Toll-Like Receptor 9 Expression With Prognosis In Breast Carcinoma. J. Contemp. Med..

[45] Moaz I., Fouad F.A., Elmasry H., Tarek G., Elzoheiry A., Elgamal M., Ibrahim R., Hisham Y., Safwat G., Kamel M.M. (2023). Associations Between Serum Soluble Toll-like Receptors 4 and 9 and Breast Cancer in Egyptian Patients. Cancer Control.

[46] Sible E., Weissman G., Amoyal S., Roblot G., Marotel M., Ainouze M., Vermare N.B., Gillet C., Michallet M.C., Caux C. (2024). Loss of TLR9 expression in breast cancer tumour cells and its role in the cell cycle. bioRxiv.

[47] Tuomela J., Sandholm J., Karihtala P., Ilvesaro J., Vuopala K.S., Kauppila J.H., Kauppila S., Chen D., Pressey C., Härkönen P. (2012). Low TLR9 expression defines an aggressive subtype of triple-negative breast cancer. Breast Cancer Res. Treat..

[48] Chandler M.R., Keene K.S., Tuomela J.M., Forero-Torres A., Desmond R., Vuopala K.S., Harris K.W., Merner N.D., Selander K.S. (2017). Lower frequency of TLR9 variant associated with protection from breast cancer among African Americans. PLoS ONE.

[49] Abdulwahid A.G., Abdullah H.N. (2020). Expression of Serum IL-22, IL-23, and TLR9 as Tumor Markers in Untreated Breast Cancer Patients. Int. J. Drug Deliv. Sci. Technol..

[50] Karki K., Pande D., Negi R., Khanna S., Khanna R.S., Khanna H.D. (2014). Expression of serum toll-like receptor 9 and oxidative damage markers in benign and malignant breast diseases. DNA Cell Biol..

[51] Shi S., Xu C., Fang X., Zhang Y., Li H., Wen W., Yang G. (2020). Expression profile of Toll-like receptors in human breast cancer. Mol. Med. Rep..

[52] Sato Y., Goto Y., Narita N., Hoon D.S. (2009). Cancer Cells Expressing Toll-like Receptors and the Tumor Microenvironment. Cancer Microenviron..

[53] Parroche P., Roblot G., Le Calvez-Kelm F., Tout I., Marotel M., Malfroy M., Durand G., McKay J., Ainouze M., Carreira C. (2016). TLR9 re-expression in cancer cells extends the S-phase and stabilizes p16^INK4a^ protein expression. Oncogenesis.

[54] Gao C., Qiao T., Zhang B., Yuan S., Zhuang X., Luo Y. (2018). TLR9 signaling activation at different stages in colorectal cancer and NF-kappaB expression. OncoTargets Ther..

[55] Resler A.J., Malone K.E., Johnson L.G., Malkki M., Petersdorf E.W., McKnight B., Madeleine M.M. (2013). Genetic variation in TLR or NFkappaB pathways and the risk of breast cancer: A case-control study. BMC Cancer.

[56] Shahzad N., Shuda M., Gheit T., Kwun H.J., Cornet I., Saidj D., Zannetti C., Hasan U., Chang Y., Moore P.S. (2013). The T antigen locus of Merkel cell polyomavirus downregulates human Toll-like receptor 9 expression. J. Virol..

[57] Hasan U. (2014). Human papillomavirus (HPV) deregulation of Toll-like receptor 9. Oncoimmunology.

[58] Pacini L., Savini C., Ghittoni R., Saidj D., Lamartine J., Hasan U.A., Accardi R., Tommasino M. (2015). Downregulation of Toll-Like Receptor 9 Expression by Beta Human Papillomavirus 38 and Implications for Cell Cycle Control. J. Virol..

[59] Tout I., Gomes M., Ainouze M., Marotel M., Pecoul T., Durantel D., Vaccarella S., Dubois B., Loustaud-Ratti V., Walzer T. (2018). Hepatitis B Virus Blocks the CRE/CREB Complex and Prevents TLR9 Transcription and Function in Human B Cells. J. Immunol..

